# Supplementary material for: An intricate functional relationship between NuA4 and Sfp1 regulates ribosome biogenesis in response to nutrient availability
Source: J Biol Chem. 2026 May 6;302(6):113107. doi: 10.1016/j.jbc.2026.113107 (PMC13254600; doi:10.1016/j.jbc.2026.113107)
Supplement: Supporting Figures S1–S7 and Table S1 [file mmc1.pdf]

SUPPLEMENTAL FIGURE LEGENDS

Figure S1. NuA4 binding at RP gene promoters is not affected by Sfp1 depletion. A-B) Loss of Sfp1-FRB at *RPL2B* and *RPS11B* gene promoters upon anchor-away treatment determined by ChIP-qPCR using an anti-FRB antibody**.** Sfp1-FRB or untagged (no-FRB) cells were treated with rapamycin (1 μg/mL) for 2 hours. Error bars represent the range of two independent experiments. **C-D)** ChIP-qPCR showing the binding of the NuA4 complex (Epl1 and Eaf1 subunits) at the promoters of *RPL2B* and *RPS11B*. A *SFP1*-deleted strain shows no significant change in NuA4 binding compared to the wild-type strain. Values are a ratio of input (%). Error bars in D are the standard error of the values (*n*=3). **E)** The Gal-induced Sfp1 overexpression plasmid (HA-tagged Sfp1) was constructed and transformed into the strain expressing endogenous myc-tagged Sfp1. Sfp1 protein levels were determined by western blotting before and after overexpression. Endogenous Myc-tagged Sfp1 shows no protein level change in raffinose and galactose. The Gal-induced Sfp1 overexpression was detected using the anti-HA antibody. **F)** ChIP-qPCR signal showing Eaf1 binding at RP genes (*RPL2B* and *RPS11B*) promoters is not affected upon Sfp1 overexpression. Values are ratio of input and error bars are the range of two independent experiments. **G)** Metagene analysis of CUTnRUN-seq signals (read counts) for Sfp1 (duplicates) and Eaf1 (NuA4) around the transcription start sites (TSS) of genes. H3K4me3 signals were also mapped in comparison (dark green). **H)** Venn diagram of overlap between Sfp1 and NuA4 peaks determined by CUTnRUN-seq.

Figure S2. Histone acetylation and Htz1 occupancy changes upon Sfp1 depletion. A) Heatmaps showing ChIP-seq of H3K9ac, H4K8ac and Htz1 occupancy in normal and Sfp1-depleted

conditions. An anti-H3 antibody was used as control to correct for total histone occupancy. Signals for a window of -400 to +400 bp relative to TSS are displayed. **B)** ChIP-qPCR of H4K8ac at the promoters of selected RP (*RPS11B, RPL23B*) and RiBi (*HAS1, NOB1*) genes confirming decreases after Sfp1 depletion. Signals are presented as a ratio on total H3 to correct for nucleosome occupancy. Error bars are the range from two biological replicates.

Figure S3. Sfp1 affects histone variant Htz1 occupancy and its acetylation. A) ChIP-qPCR of Htz1 at the promoters of selected RP (*RPS11B*) and RiBi (*HAS1, NOB1*) genes confirming increases after Sfp1 depletion. Signals are presented as a ratio on total H4 to correct for nucleosome occupancy. Error bars are the range from two biological replicates. **B)** Htz1 acetylation is decreased in Sfp1-depleted cells. Immunoprecipitations (IP) were performed with whole cell extracts from cells depleted or not for Sfp1 using an anti-acetyl-lysine antibody. Htz1, H4 and H3 acetylation levels were determined by western blotting. **C**) Western blot analysis of whole cell extracts from wild-type and *sfp1* deleted cells. Bulk histone H4 acetylation, Htz1 and Htz1 acetylation levels are shown. Pgk1 signal is shown as loading control. **D)** Venn diagram showing the overlap between our wild-type Sfp1 CUT&RUN peaks (Fig 3) and published Htz1 ChIP-seq peaks.

Figure S4. K655/657 mutants show almost no effect on Sfp1 protein expression and slight growth defect in YPD. A) Expression of Sfp1 proteins in wild-type and mutated strains. Pgk1 is a reference protein. **B)** The indicated strains were grown to the log phase in YPD. Growth curves are depicted at representative time points. Error bars are standard deviation from three different cultures. **C)** Venn diagram showing the overlap between wild-type and Q mutant Sfp1

CUT&RUN peaks (Fig 4). **D)** IGV tracks showing Sfp1 CUT&RUN peaks at the selected *HAS1* gene (RiBi) promoter.

Figure S5. Principal component analysis (PCA) of the RNA-Seq samples. The PCA figure demonstrates variance between RNA-seq samples. Wild type and mutated samples (duplicates) at different time points are represented by different colours, as indicated.

Figure S6. Genes involved in metabolic processes are downregulated in the Sfp1 K655/657Q strain. Gene set enrichment analysis (GSEA) of RNA-seq data was performed on wild-type cells and acetyl-mimic Sfp1 cells growing in YPD. Top up- and down-regulated pathways are shown.

Figure S7. Sfp1 regulates RiBi and RP gene expression through different mechanisms upon glucose pulse after the shift in carbon source. A) RNAs were extracted from log phase wild-type Sfp1, K655/657R, and K655/657Q strains grown in indicated times of glucose pulse. Gene expression levels of selected RiBi genes (*NSA1* and *RPF2*) were quantified, relative to levels of *ACT1* mRNA, by RT-qPCR. Values were normalized to time 0 in each strain. Error bars represent the range of two independent experiments. **B)** Volcano plots showing differential gene expression analysis by RNA-seq of cells expressing wild-type Sfp1 and K655/657Q mutation. Expression changes at different time points upon glucose pulse after carbon source shift are shown. RP genes are highlighted.

Supplemental Table 1 Yeast strains used in this study.

Strain	Genotype	Reference
BY4741	*MATα leu2Δ0 ura3Δ0 his3-1 met15Δ0*	Euroscarf
LPY3431	*MATa his3Δ200 leu2-3,112 trp1Δ1 ura3-52*	Clarke *et al.*, Mol Cell Biol 1999
W303-1A	*MATa ade2-1 his3-11,15 leu2-3,112 can1-100 trp1-1 ura3-1*	Thomas and Rothstein, Cell 1989
JKM139	*MATa hoΔ hmlΔ::ADE1 hmrΔ::ADE1 ade1-100 leu2-3,112 trp1::hisG' lys5 ura3-52 ade3::GAL::HO*	Lee *et al.*, Cell 1998
QY708	LPY3431 *EPL1-TAP::TRP1*	Rossetto *et al.*, EMBO J 2014
QY3836	W303-1A *SFP1-TAP::TRP1*	this study
QY3530	JKM139 *fpr1Δ*::HphMX *tor1-1::URA3 RPL13A-2*FKBP12::TRP1* (No-FRB)	Cheng *et al.*, PLoS Genet 2021
QY3572	QY3530 *FRB-ESA1::KanMX*	Cheng *et al.*, PLoS Genet 2021
QY3854	QY3530 *SFP1-FRB::KanMX*	this study
QY3856	QY3530 *SFP1-FRB::KanMX EPL1-3FLAG::NatRMX*	this study
QY3843	W303-1A *sfp1Δ::TRP1*	this study
QY3844	W303-1A *EPL1-13MYC::KanMX*	this study
QY3845	W303-1A *sfp1Δ::TRP1 EPL1-13MYC::KanMX*	this study
QY3847	W303-1A *SFP1-K655R-K657R* (CRISPR)	this study
QY3848	W303-1A *SFP1-K655Q-K657Q* (CRISPR)	this study
QY3849	LPY3431 *SFP1-13MYC::KanMX*	this study
QY3851	LPY3431 *SFP1-K655R-K657R-13MYC::KanMX*	this study
QY3852	LPY3431 *SFP1-K655Q-K657Q-13MYC::KanMX*	this study
QY3853	W303-1A *SFP1-13MYC::KanMX* + pBG1805(*GAL-SFP1-6HIS-HA*)	this study
YMD070	*MATα leu2Δ0 ura3Δ0 his3-1 met15Δ0 sir2Δ::HYGMX hst1Δ::KANMX hst2Δ::HIS3MX*	Rössl *et al.*, Genetics 2016
QY3858	YMD070 *SFP1-13MYC::LEU2*	this study
QY3862	YMD070 SFP1- *K655R-K657R-13MYC::LEU2*	this study
QY3863	YMD070 SFP1- *K655Q-K657Q-13MYC::LEU2*	this study

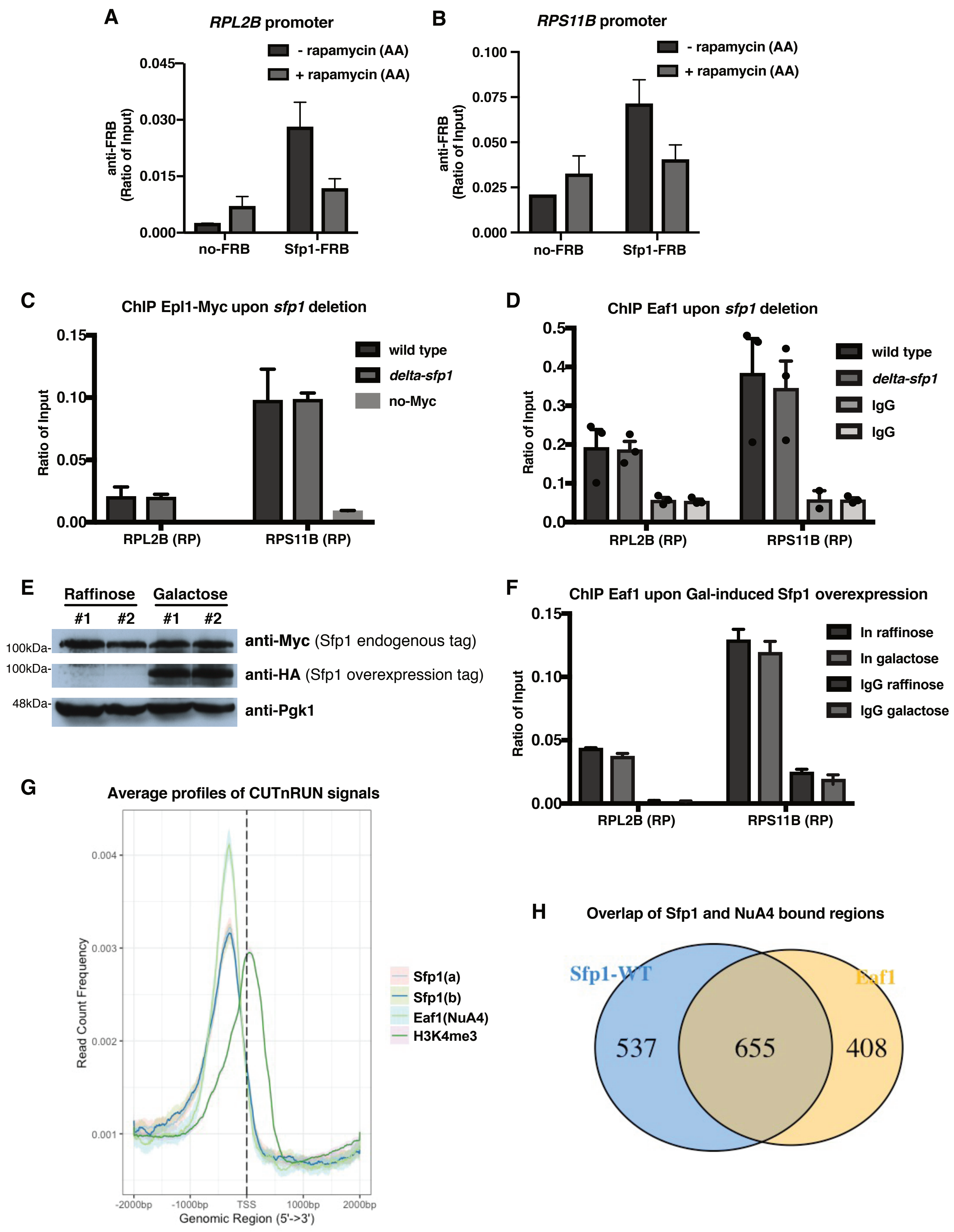
A
RPL2B promoter
- rapamycin (AA)
+ rapamycin (AA)
anti-FRB (Ratio of Input)
0.045
0.030
0.015
0.000
no-FRB
Sfp1-FRB
B
RPS11B promoter
- rapamycin (AA)
+ rapamycin (AA)
anti-FRB (Ratio of Input)
0.100
0.075
0.050
0.025
0.000
no-FRB
Sfp1-FRB
C
ChIP Epl1-Myc upon sfp1 deletion
wild type
delta-sfp1
no-Myc
Ratio of Input
0.15
0.10
0.05
0.00
RPL2B (RP)
RPS11B (RP)
D
ChIP Eaf1 upon sfp1 deletion
wild type
delta-sfp1
IgG
IgG
Ratio of Input
0.5
0.4
0.3
0.2
0.1
0.0
RPL2B (RP)
RPS11B (RP)
E
Raffinose
Galactose
#1
#2
#1
#2
100kDa-
100kDa-
48kDa-
anti-Myc (Sfp1 endogenous tag)
anti-HA (Sfp1 overexpression tag)
anti-Pgk1
F
ChIP Eaf1 upon Gal-induced Sfp1 overexpression
In raffinose
In galactose
IgG raffinose
IgG galactose
Ratio of Input
0.15
0.10
0.05
0.00
RPL2B (RP)
RPS11B (RP)
G
Average profiles of CUTnRUN signals
Read Count Frequency
0.004
0.003
0.002
0.001
-2000bp
-1000bp
TSS
1000bp
2000bp
Genomic Region (5'->3')
Sfp1(a)
Sfp1(b)
Eaf1(NuA4)
H3K4me3
H
Overlap of Sfp1 and NuA4 bound regions
Sfp1-WT
Eaf1
537
655
408

A

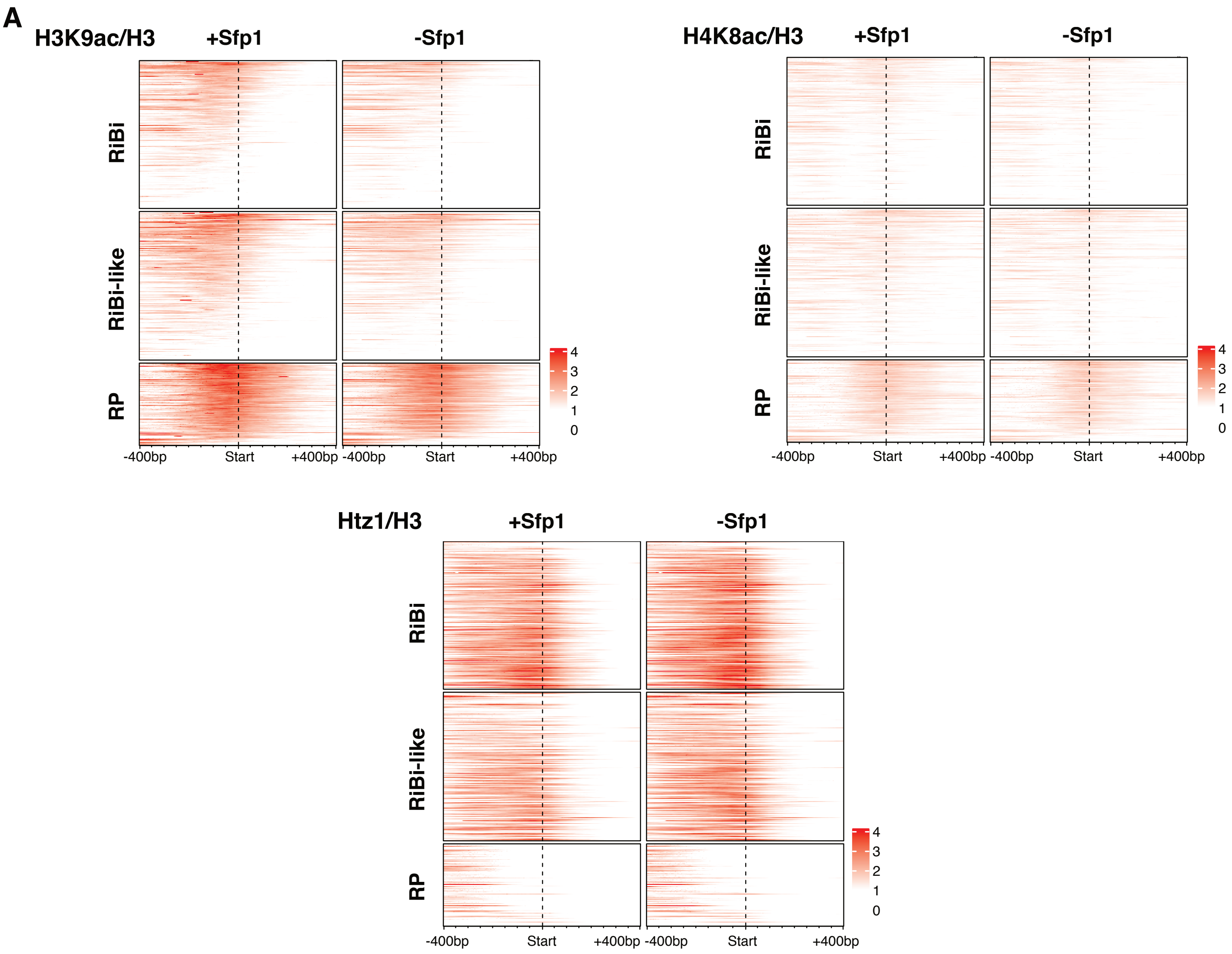
H3K9ac/H3
+Sfp1
-Sfp1
RiBi
RiBi-like
RP
4
3
2
1
0
-400bp
Start
+400bp
H4K8ac/H3
Htz1/H3

B

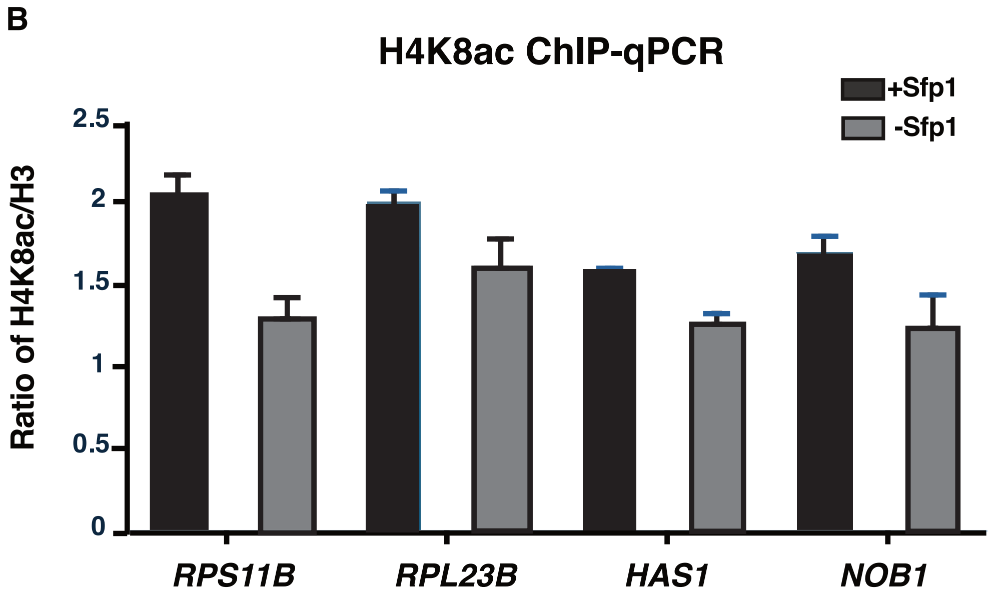
H4K8ac ChIP-qPCR
+Sfp1
-Sfp1
Ratio of H4K8ac/H3
2.5
2
1.5
1
0.5
0
RPS11B
RPL23B
HAS1
NOB1

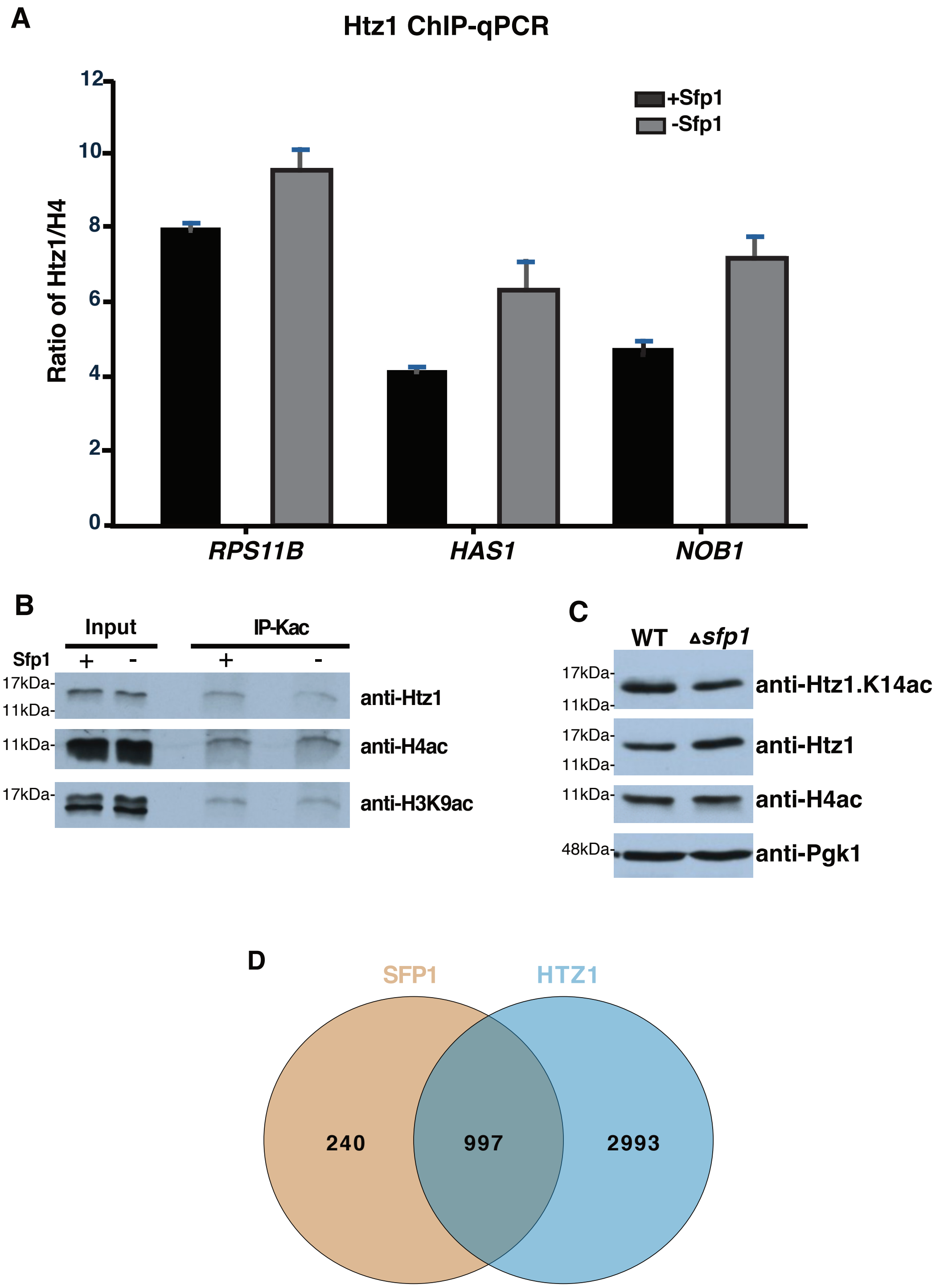
A
Htz1 ChIP-qPCR
Ratio of Htz1/H4
0
2
4
6
8
10
12
+Sfp1
-Sfp1
RPS11B
HAS1
NOB1
B
Input
IP-Kac
Sfp1
+
-
+
-
17kDa-
11kDa-
anti-Htz1
11kDa-
anti-H4ac
17kDa-
anti-H3K9ac
C
WT
Δsfp1
17kDa-
11kDa-
anti-Htz1.K14ac
17kDa-
11kDa-
anti-Htz1
11kDa-
anti-H4ac
48kDa-
anti-Pgk1
D
SFP1
HTZ1
240
997
2993

Xu et al. Fig S4

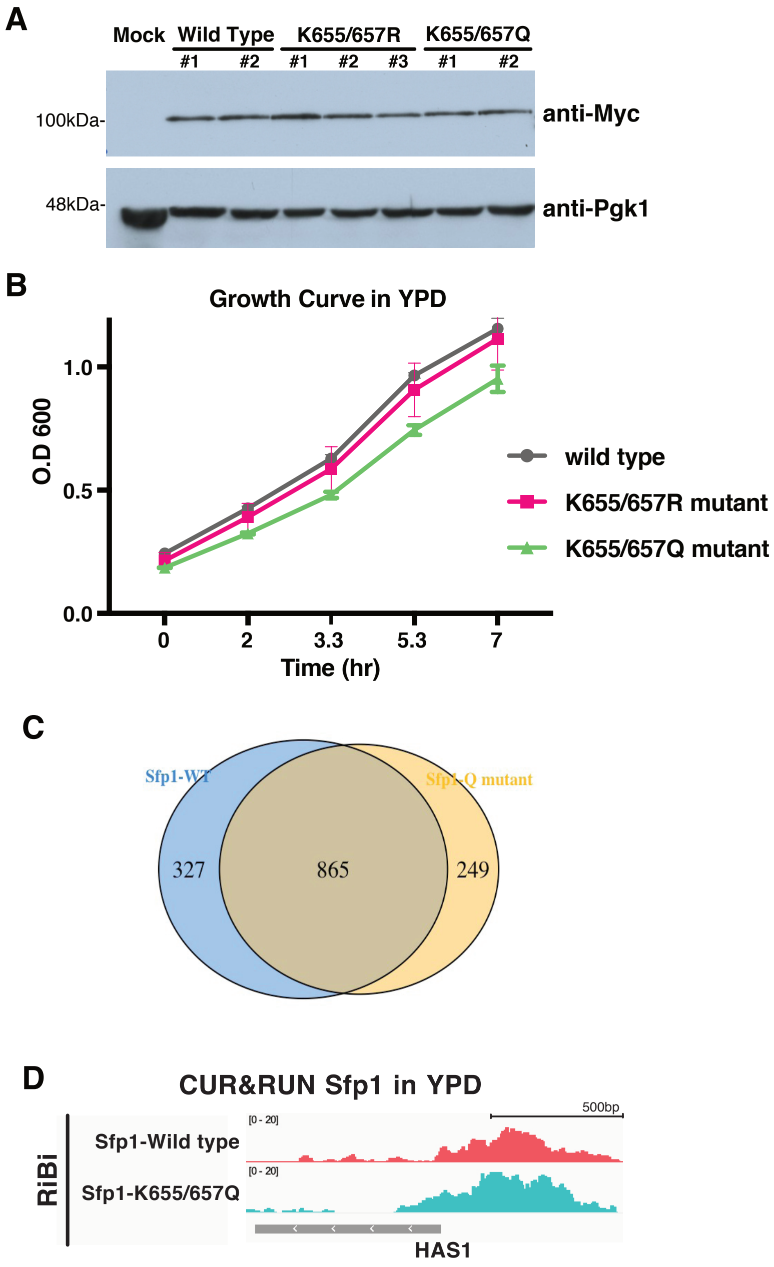

Xu et al. Fig S5

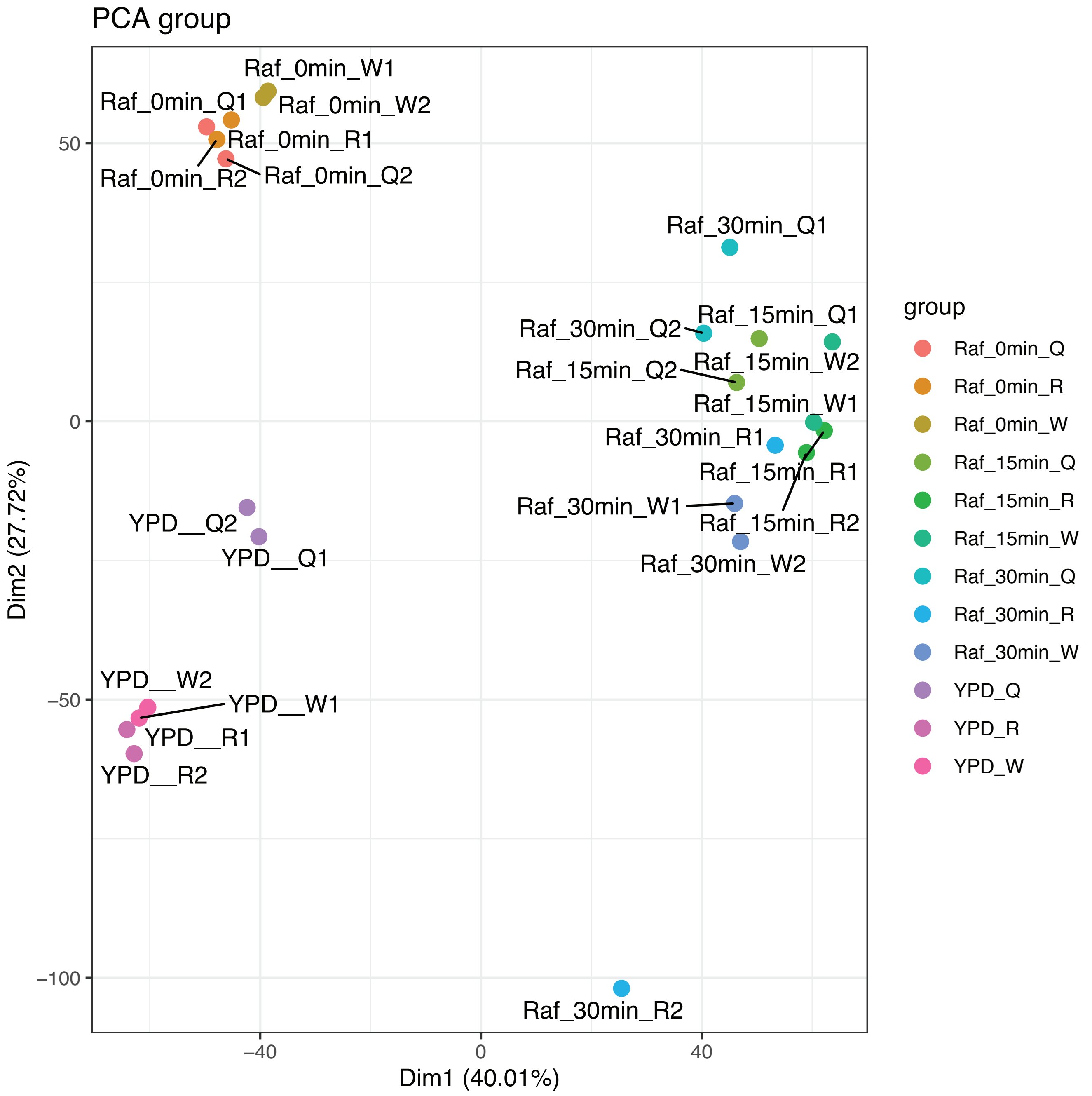

Xu et al. Fig S6

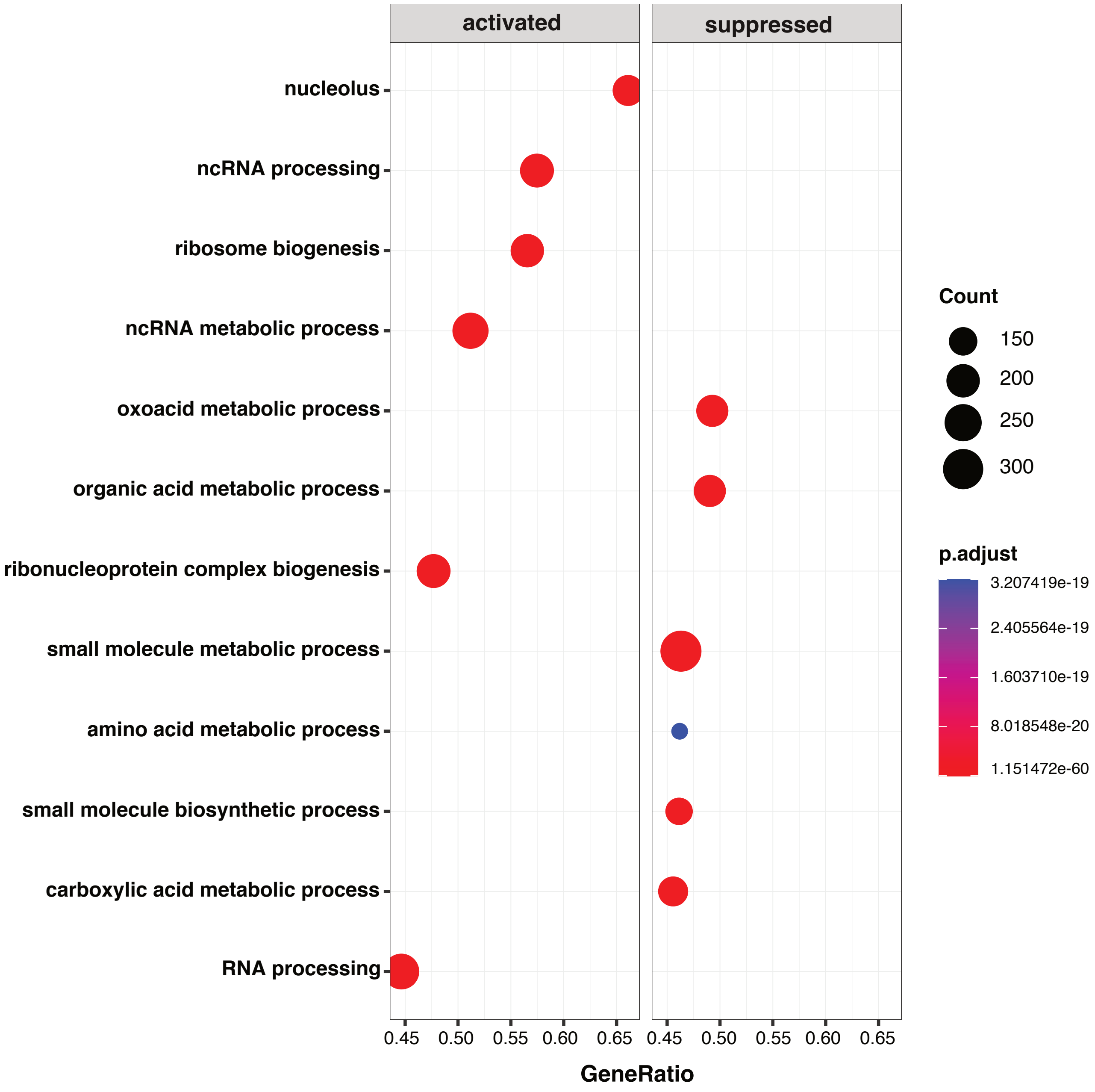

A

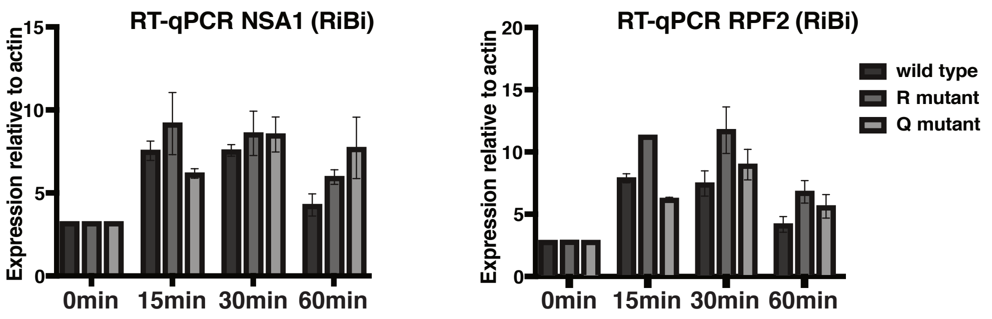
RT-qPCR NSA1 (RiBi)
RT-qPCR RPF2 (RiBi)
Expression relative to actin
0min
15min
30min
60min
wild type
R mutant
Q mutant

B

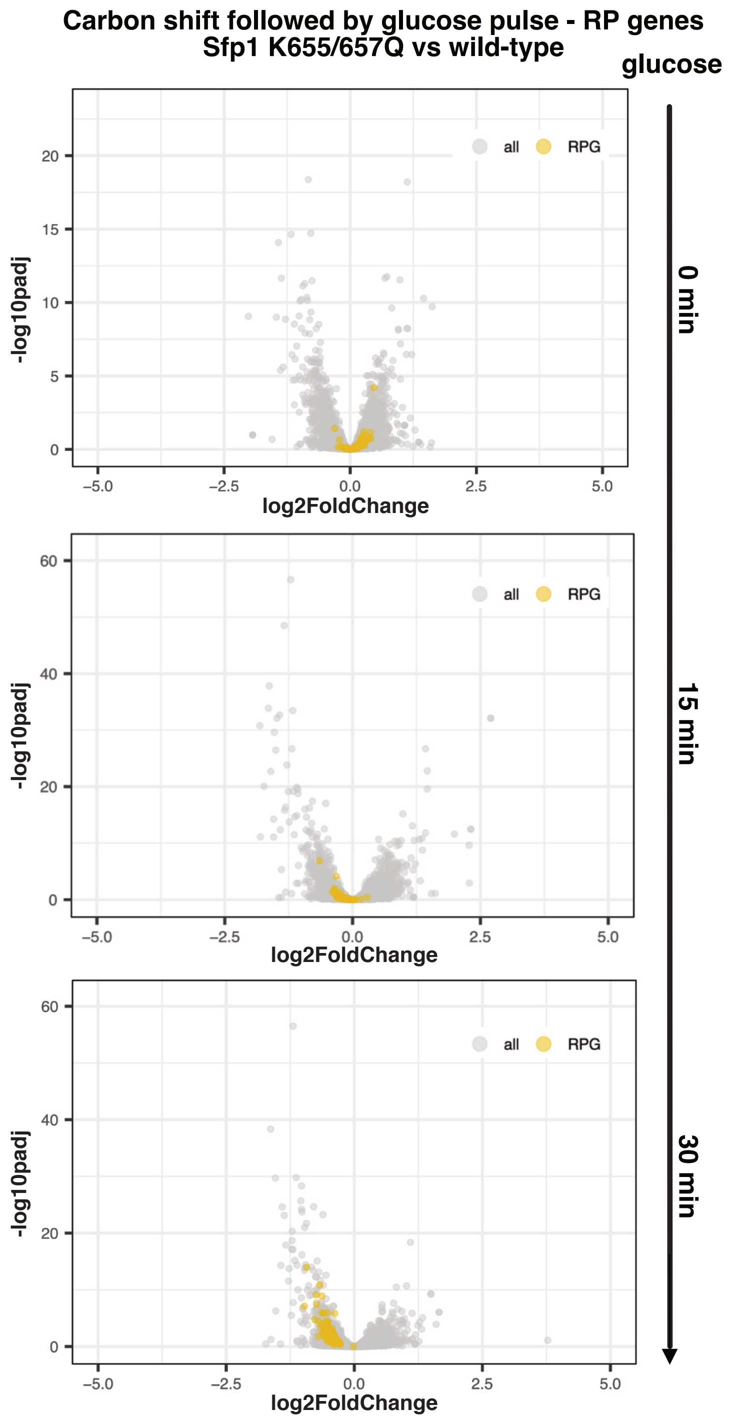
Carbon shift followed by glucose pulse - RP genes
Sfp1 K655/657Q vs wild-type
glucose
all
RPG
-log10padj
log2FoldChange
0 min
15 min
30 min
